# Prescription of antibiotics in dentistry - a report from the Swedish STRAMA work

**DOI:** 10.1080/20002297.2017.1325230

**Published:** 2017-05-30

**Authors:** Gunnar Dahlen

**Affiliations:** ^a^ Oral Microbiology and Immunology, Inst of Odontology, Sahlgrenska Academy, University of Gothenburg, Sweden

## Abstract

Overuse of antibiotics is today a global concern. National programs, programs within EU and WHO against misuse of antibiotics have been created. Swedish strategic program against antibiotic resistance (STRAMA) was initiated in 1997, within the Swedish Public Health Agency (SPHA) and consisted of volunteers from various medical disciplines to work for a reduction of antibiotic prescription. A National Dentistry Section of STRAMA was established in 2007 affiliated to SPHA. An annual update on the prescription pattern among Swedish dentists is now available.

A decrease of antibiotic prescription in both medicine and dentistry the last 10-15 years has been noticed. Dentists prescribe 7% of all antibiotics of which 78% was in 2009 penicillin V, 10% clindamycin, and 9% amoxicillin. The total prescriptions among dentists have decreased from 35 per 1000 inhabitants and year in 2007 to 23 in 2016. Between 2012-2014 the prescription rate for amoxicillin decreased 30%, probably due to new guidelines for prophylaxis. There is still a significant difference in the prescription pattern between dentists in greater cities and rural provinces. The work continues to inform students and dentists on erroneous or unnecessary prescription by updated guidelines and recommendations.

